# Dietary Amino Acids Promote Glucagon-like Hormone Release to Generate Novel Calcium Waves in Adipose Tissues

**DOI:** 10.21203/rs.3.rs-4493132/v1

**Published:** 2024-06-12

**Authors:** Li He, Muhammad Ahmad, Shang Wu, Shengyao Luo, Wenjia Shi, Xuan Guo, Yuansheng Cao, Norbert Perrimon

**Affiliations:** University of Science and Technology of China; Harvard Medical School; University of Science and Technology of China; Peking University; Xi’an University of Technology; Jinzhou Medical University; Tsinghua University; Harvard Medical School

## Abstract

Nutrient sensing and the subsequent metabolic responses are fundamental functions of animals, closely linked to diseases such as type 2 diabetes and various obesity-related morbidities. Among different metabolic regulatory signals, cytosolic Ca^2+^ plays pivotal roles in metabolic regulation, including glycolysis, gluconeogenesis, and lipolysis. Recently, intercellular calcium waves (ICWs), the propagation of Ca^2+^ signaling through tissues, have been found in different systems to coordinate multicellular responses. Nevertheless, our understanding of how ICWs are modulated and operate within living organisms remains limited. In this study, we explore the real-time dynamics, both in organ culture and free-behaving animals, of ICWs in *Drosophila* larval and adult adipose tissues. We identified Adipokinetic hormone (AKH), the fly functional homolog of mammalian glucagon, as the key factor driving Ca^2+^ activities in adipose tissue. Interestingly, we found that AKH, which is released in a pulsatile manner into the circulating hemolymph from the AKH-producing neurosecretory cells (APCs) in the brain, stimulates ICWs in the larval fat by a previously unrecognized gap-junction-independent mechanism to promote lipolysis. In the adult fat body, however, gap-junction-dependent random ICWs are triggered by a presumably uniformly diffused AKH. This highlights the stage-specific interplay of hormone secretion, extracellular diffusion, and intercellular communication in the regulation of Ca^2+^ dynamics. Additionally, we discovered that specific dietary amino acids activate the APCs, leading to increased intracellular Ca^2+^ and subsequent AKH secretion. Altogether, our findings identify that dietary amino acids regulate the release of AKH peptides from the APCs, which subsequently stimulates novel gap-junction-independent ICWs in adipose tissues, thereby enhancing lipid metabolism.

## Introduction

Adipose tissue is a primary organ of fat storage that coordinates energy metabolism pathways under various nutritional states. Such metabolic processes are under the hormonal control of insulin and glucagon, the two key hormones playing a central role in energy storage and homeostasis regulation. Notably, glucagon promotes lipid breakdown in the adipose tissue by elevating cytoplasmic Ca^2+^ levels through activation of its GPCR receptor^1^. Intriguingly, these Ca^2+^ activities are spatially organized into intercellular Calcium waves (ICWs) across the mammalian liver^2^. However, the mechanism and biological significance of this collective ICWs *in vivo* remains poorly understood. Fruit flies have a functional homolog of glucagon, namely Adipokinetic hormone (AKH), which plays similar roles to mobilize adipose lipids upon starvation^3–5^. In this study, we demonstrated that AKH triggers significant ICWs in the fly adipose tissue, presenting a unique opportunity to investigate the mechanics and regulations of ICWs *in vivo*.

ICWs are complex tissue-level biological events that exist in a wide array of cell types in multicellular organisms and that impact many biological processes relevant to cellular life (e.g., muscle contraction^6,7^, gene expression^8,9^, cellular proliferation^10^, differentiation^11^, neuronal firing and excitability^12^, and metabolism^13^). Dysregulated Ca^2+^ signaling can result in various disease states ranging from various neurological disorders to cardiovascular diseases and cancer^14,15^. ICWs are generated by an increase of cytoplasmic Ca^2+^ ions concentration from organelle reserves as well as propagation of the signal from one cell to its neighbors, which consequently coordinate concerted action at the tissue level. The propagation of ICWs has been reported to be governed by two mechanisms: (1) the direct diffusion of the secondary messengers i.e. Ca^2+^ and IP_3_ through gap junctions that stimulate Ca^2+^ efflux in neighboring cells^16,17^; and (2) paracrine signaling, where the secretion of extracellular ATP stimulates nearby cells to release Ca^2+^ from the endoplasmic reticulum (ER)^18^. Despite these insights, the underlying biophysical mechanisms and the biological significance of the emergent pattern and signal propagation of ICWs in different biological systems have largely remained unidentified.

Here, using the *Drosophila* larval fat body/adipose tissue as a model to study ICWs *in vivo*, we analyzed the real-time dynamics of ICWs by live-cell imaging. We show that novel global ICWs are triggered by AKH in the larval fat body, reminiscent of glucagon-induced Ca^2+^ waves observed in the human liver^1^. More interestingly, genetic perturbation of gap junctions significantly abrogates ICWs triggered by AKH in cultured fat bodies, indicating that these cellular junctions are required to propagate the ICWs. However, disruption of gap junctions did not affect the global propagation of waves at the tissue level, leading us to propose the existence of a gap junction-independent mechanism that helps propagate ICWs across the tissue. To characterize this mechanism, we used a combination of experimental and computational modeling approaches. Specifically, we determined that these gap-junction-independent ICWs are regulated through facilitated extracellular diffusion of AKH. Indeed, global wave propagation requires a constant circulation of hemolymph which depends on cardiac pumping, as disrupting larval lymph circulation completely abrogates the tissue-level wave propagation. Meanwhile, the global ICWs were triggered by the periodic release of AKH from the brain corpora cardiaca AKH-producing neurosecretory cells (APCs), as silencing the APCs immediately stops the ICWs in the fat body. This finding is further supported by computational simulation of the ICWs that produces similar patterns and outcomes resembling our *in vivo* observations.

Finally, we utilized this system to identify the dietary factor regulating the AKH-dependent ICWs. Previous studies have demonstrated that AKH, like its functional mammalian homolog Glucagon, plays a pivotal role in maintaining blood sugar homeostasis and modulating starvation-induced hyperactivity in adult. However, compared to insulin, the regulation of AKH remains less understood. Various dietary, hormonal and neuronal factors regulating insulin signaling have been identified in the fly^19^. Notably, it has been recently demonstrated that fly insulin-producing cells directly sense the dietary amino acid leucine, which consequently regulates the secretion of dILPs^20^. In contrast, whether specific amino acids exert regulatory control over AKH secretion remains unknown. In this study, we found that secretion of AKH from the APCs is not only repressed by sugar, but also promptly stimulated within minutes after consumption of particular amino acids and that amino acids consumption promotes fat loss in both larvae and adult flies through AKH-dependent signaling. Collectively, our study suggests that specific amino acids regulate AKH/Glucagon signaling, which in turn activates ICWs throughout adipose tissues, coordinating lipid metabolism.

## Materials and Methods

### Fly husbandry.

Flies were raised on standard food (2000 ml water, 12.8 g agar, 80 g yeast, 112 g cornmeal, 176 g glucose, 2.5 g methylparaben, 20 ml propionic acid, total 2 l of fly food) at 25°C with 12 h:12 h light: dark cycles. Fly strains used in this study are listed in **Supplementary Table 1**.

### Ex vivo GCaMP imaging in APCs.

Early 3rd instar larval brains were dissected in modified basal hemolymph-like solution devoid of any amino acids (modified HL6(AA-) buffer) (74.2 mM NaCl, 2.0 mM MgCl_2_, 10.0 mM NaHCO_3_, 24.8 mM KCl, 0.5 mM CaCl_2_, 80 mM Trehalose, 5 mM BES, pH 7.2). Dissected brains were immobilized using a holder in the perfusion chamber. The samples in the HL6 (AA-) buffer were recorded for 1 min to generate a baseline. Next, the solutions were changed to HL6 (AA-) buffer + AA (5 mM) with the pH adjusted back to 7.2 by gentle perfusion for 10 min. All imaging studies were performed with a Leica M205 FCA high-resolution stereo fluorescence microscopy (Leica).

### Ex vivo imaging of larval fat bodies.

The *ex vivo* imaging chambers were assembled on a live cell imaging dish (Nest, 801001), a metal ring with an inner diameter of 10 mm, and a cellulose film from a tea bag modified to match the chamber. Briefly, the fat bodies of 3rd instar larvae were dissected in Schneider’s medium (Sigma) and placed at the center of the imaging dish in 20 μl of Schneider’s medium. Next, the modified film was placed on top of the fat body and the metal ring was gently placed on the lm to trap the tissue underneath. Finally, 200 μl of Schneider’s medium was added inside the insert. The samples in Schneider’s medium were recorded for 5 min (time interval:5s) to generate a baseline. Then, Schneider’s medium was replaced by Schneider’s medium containing 100 ng/mL of different synthesized *Drosophila* neuropeptides (Genscript, the sequences of the peptides are shown in **Supplementary Table 2**), and recording was done for 20 min (time interval:5s). Time-lapse recording was performed on a Leica DMi 8 equipped with a Leica DFC9000 sCMOS camera and a 1.25x HC PL Fluotar objective (Leica). For higher resolution of the gap junction knockdown experiments, a 5x N Plan objective (Leica) was used. GCaMP5G was excited with a 475 laser. Imaging was performed in a dark room at 18°C. We used MATLAB to remove background noise and highlight the edges of the sample.

### Ex vivo imaging of adult fat bodies.

For adult flies, as most of the fat bodies adhere to the inside of the abdominal cavity, we dissected the dorsal shell together with the fat bodies. The dorsal shell was then adhered to a live cell imaging dish by Vaseline, such that the fat bodies face upwards, and bathed in 200 uL Schneider’s medium. Time-lapse recording was performed on a Leica M205 FCA high-resolution stereo fluorescence microscope (Leica) equipped with a Leica DFC7000 GT camera. The remaining steps and parameters are similar to those in the larval experiment.

### In vivo imaging of immobilized larvae and adults.

For larvae, we attached two coverslips to a microscope slide with double-sided tape to form a thin slit, then used plasticine to plug both sides of the slit so that the gap in the slit is the same width as a 3rd instar larva. Next, a coverslip was added on the top of the chamber to immobilize the larva. For adults, the six legs of an adult female fly were cut off, and the wings adhered to a live cell imaging dish with Vaseline so that the abdomen faced upward. For the carbon dioxide and chloroform treatment, the chamber was covered with a transparent petri dish to prevent gas leakage. Time-lapse recording was performed on a Leica M205 FCA high-resolution stereo fluorescence microscope (Leica) equipped with a Leica DFC7000 GT camera.

### In vivo imaging of fat bodies of free-behaving larvae.

We used a 3D printed mold (Wenext) to produce an agarose gel containing different nutrients. The center of the gel has a 11mm*13mm*0.66mm chamber, which can provide a free-behaving arena for more than 10 early 3rd instar larvae. Finally, a coverslip was added on top of the chamber to prevent the larvae from escaping. After being allowed to acclimate for 10 min, larvae were recorded for 20 min (time interval:5s), then all larvae were transferred to an agarose gel chamber containing another type of food for 20-min recording. To ensure that there was no food residue in and on the body of the 3rd instar larvae, larvae were starved for 9 hours before imaging and washed with ddH_2_0 during each transfer process. Time-lapse recording was performed on a Leica M205 FCA high-resolution stereo fluorescence microscope (Leica) equipped with a Leica DFC7000 GT camera. In image processing, we used connected component analysis to approximate each connectome as a larva. Fluorescent signal changes were normalized using the following formula: ΔF/F_0_ = (F(t) – F_0_)/F_0_, where F(t) is the uorescence at time t, F_0_ is the average baseline (before transfer).

### In vivo imaging of APCs of free-behaving larvae.

To perform high-resolution neuronal imaging of free-behaving 1st instar larvae, we used an extended-depth-of-field microscope with two modules: 1. A darkfield imaging module equipped with a 4X NA 0.2 air objective (Nikon, Japan) and a high-speed near-infrared camera (acA2000–340kmNIR, Basler ace), used to track and record a free-behaving 1st instar larva. 2. A fluorescence imaging module equipped with a 10X NA 0.3 air objective and a sCMOS camera (Zyla 4.2, Andor Inc., UK), with an imaging surface split in two by Optosplit II (Cairn, UK), which allows simultaneous recording of two fluorescent signals (calcium-sensitive GCaMP and calcium-insensitive RFP used as reference). Through extended-depth-of-field technology, the effective depth of field was extended by about 5 times, avoiding errors on the Z-axis caused by motion. Before imaging, the 1st instar larvae were starved for 6 hours, and then the larvae were gently picked with a brush into an agarose gel chamber (Φ20 mm*0.15 mm) made with a 3D printed mold (Wenext) for 15-min recording. All image analyses were conducted using ImageJ and MATLAB. The fluorescent signal changes were normalized using the following formula: ΔF/F_0_ = (F(t) – F_0_)/F_0_, where F(t) is the fluorescence at time t, F_0_ is the average baseline (before transfer).

### AKH secretion assay.

To measure AKH retention in PACs, early 3rd instar larvae were picked out from standard food and washed with ddH_2_0, and after feeding for a period of time under different dietary conditions, the brains were dissected in PBS (1.86 mM NaH_2_PO_4_, 8.41 mM Na_2_HPO_4_, 175 mM NaCl) and fixed in 4% v/v paraformaldehyde (PFA)/PBS for 30 min at 23°C. After washing in PBST (PBS + 0.05% Triton X-100, BBI) (3 times, 10 min each), the brains were blocked in 5% BSA (Solarbio, A8020) in PBST for 1 h at 25°C, then incubated with rabbit anti-AKH (1:1000; ABclonal, WG-05853) for 12–20 h at 4°C. After washing in PBST (3 times, 10 min each), the sample brains were incubated with Alexa Fluor 488 goat anti-rabbit IgG (1:500; Invitrogen) for 1 h at 25°C and washed again using PBST (3 times, 10 min each). We used an antifade agent to mount the samples. All images were acquired using a Leica M205 FCA high-resolution stereo fluorescence microscope (Leica).

### Wave direction analysis.

We set the direction of the larvae from head to tail as columns, took the average of each row, and derived a one-dimensional vector for each frame. Next, we correlated these measurements with time to obtain a two-dimensional graph to show the direction of calcium wave movement.

### Ca^2+^ proportion calculation.

We use the baseline fluorescence of GCaMP5G as the threshold to calculate the proportion of the area where calcium activation occurs. Each calculation requires at least 20 minutes of time-lapse data. This parameter also represents the probability of a calcium activation event occurring per unit time per unit area.

### Wave velocity calculation.

We first defined two lines parallel to the wavefront. Then, we measured the time required by the wavefront to cross the distance between the two lines to manually calculate wave velocity using ImageJ.

### Ca^2+^ diffusion calculation.

To characterize calcium wave propagation, we artificially set a parameter “Ca^2+^ diffusion area”. According to the concept of connected components in image processing, we calculated the area of each connected region where calcium activation occurred in the 20-min time-lapse data and calculated the average value.

### TAG assay.

5 flies from each group were homogenized with 100 μl of isopropyl alcohol (BBI, A600918–0500), centrifuged at 10,000g for 10 minutes at 4°C, and the supernatant was collected. 2 μl of sample solution was mixed with 200 μl of assay reagent (Elabscience, E-BC-K261-M), and incubated at 37°C for 10 minutes. We measured the absorbance at 492 nm in a microplate reader (Thermo Scientific Multiskan FC).

### Heart rate assay.

The early 3rd instar larvae were attached to a glass slide with light-curing glue, ensuring that their dorsal sides were facing up. Recordings were captured on a Leica M205 FCA high-resolution stereo fluorescence microscope (Leica) equipped with a Leica DFC7000 GT camera. The tracheal movements can readily be seen moving with each heartbeat. Kymographs were generated by drawing a single-pixel straight line perpendicular to the trachea in each frame. All image analyses were conducted using ImageJ.

### Hemolymph ow assay.

To detect the direction of the hemolymph flow, flies anesthetized with carbon dioxide were injected with PBS + 0.1%BSA containing 5 μm diameter fluorescent beads (ex/em: 535/610 nm, Hugebio). Before the injection, the beads were blocked in PBS + 10% yeast extract overnight to prevent adhesion to the tissue *in vivo*. For larvae, we chose to inject from the tail, and for adults, we chose to inject from the abdomen. After injection, the flies were attached to a glass slide with light-curing glue. The recording was performed on a Leica M205 FCA microscope. All image analyses were conducted using ImageJ and MATLAB.

### Statistics.

Sample sizes were determined through preliminary experiments and previous studies to achieve the required statistical power. All assays were repeated more than three times. Quantitative and statistical parameters, including statistical methods, error bars, n numbers, and p-values, are indicated in each figure. Error bars shown in all results are from biological replicates. Differences were assessed using a two-tailed unpaired Student’s t-test, unless stated otherwise in the figure legends. P < 0.05 was considered statistically significant. Significance was noted as *p < 0.05, **p < 0.01, ***p < 0.001. Image processing and quantification were performed in ImageJ and MATLAB. Plotting of graphs and statistical analyses were conducted with GraphPad Prism 8.4.2.

## Results

### Periodic ICWs in fly larval adipose tissues are induced by a brain-derived factor

**Figure 1:**
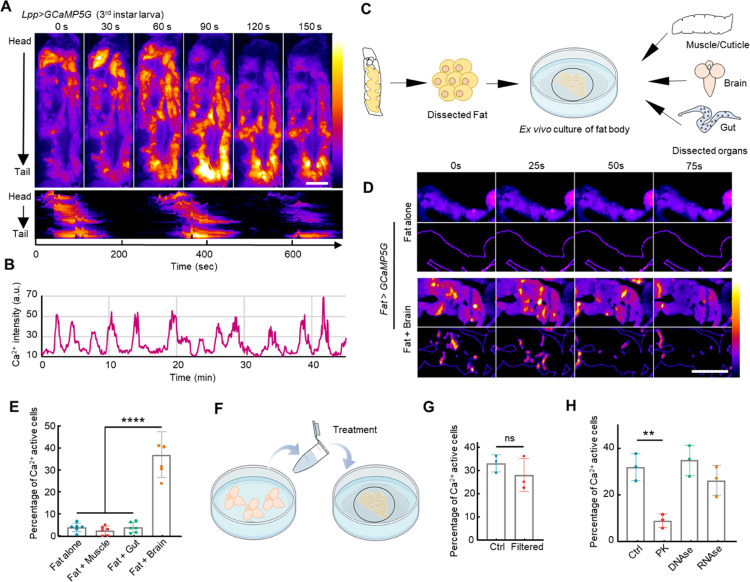
Periodic Ca^2+^ waves are triggered in the fly larval fat body by a brain-derived factor. **(A-B)** A 3^rd^ instar larva was placed in a narrow glass channel to restrict its mobility. Fat body-specific Ca^2+^ activity was visualized using *Fb-GAl4*>*UAS-GCaMP5G*. A kymograph of Ca^2+^ waves traveling from head to tail is shown. (**B**) Representative Ca^2+^ activity in the larval fat body. (**C**) Fat body expressing GCaMP5G was dissected and cultured in Schneider’s *Drosophila* medium with or without different larval organs. (**D**) Fat body cultured alone showed little Ca^2+^ activity, while fat body cultured together with dissected brains showed prominent ICWs. The dynamic Ca^2+^ activities were highlighted in the lower panel by removing the constant background signal. (**E**) Quantification of Ca^2+^ activities in cultured fat bodies under different conditions. (**F**) Brain-conditioned medium was used to treat the isolated fat bodies. (**G**) Brain-conditioned medium before and after filtration triggered significant ICWs in cultured fat bodies. (**H**) Ca^2+^ activity triggered by proteinase K-digested brain-conditioned medium was significantly reduced compared to the untreated brain-conditioned medium. Proteinase K-digested brain-conditioned medium was treated with 0.1mg/mL proteinase K for 1 hour at 37°C and then filtered with Pierce 10K MWCO concentrator. The untreated brain-conditioned medium was processed similarly without the addition of proteinase K. Data were plotted as mean ± SD. Scale bars, 500 μm (**A**), 500 μm (**D**).

The fly fat body, which is the functional equivalent of the mammalian liver and adipose tissue, plays central roles in metabolic regulation and nutrient sensing^21^. Ca^2+^ levels in the fly fat body are essential in lipolysis^22,23^, and *in vivo* Ca^2+^ waves in the larval fat body have been mentioned without in-depth study^24^. Similarly, Ca^2+^ waves have been reported in the mammalian liver^25^. Despite these findings, the functional consequences of what triggers such waves have not been investigated *in vivo* in the fly fat body. To address these questions, we expressed the genetically encoded Ca^2+^ indicator *GCaMP5G* specifically in the larval fat body, and immobilized larvae within a glass channel for observation (**Supplementary Fig. 1A**). To our surprise, we noticed global intercellular Calcium waves (ICWs) that emanate in a periodic fashion from the larval head to tail (Fig. 1A–B, **Supplementary video 1**). To ensure that these waves were not an artefact due to immobilization of the larvae, we examined Ca^2+^ activity in free-behaving 3rd instar larvae. Similarly, prominent global ICWs were observed, indicating that these waves were not a consequence of immobilization (**Supplementary Fig. 1B-C**). We examined whether the global ICWs could be caused by the contractions of skeletal muscles which may mechanically stimulate the fat body. Paralyzing the larva by feeding with neurotoxin Tetrodotoxin (TTX) completely stopped muscle movements, but the global ICWs persisted, suggesting the ICWs are not caused by muscle contractions (**Supplementary Fig. 2**).

Previous studies in fly imaginal discs suggested that ICWs are triggered autonomously or in response to self-secreted epithelial-derived factors^26,27^. To discern whether the Ca^2+^ in the fat body is autonomously regulated or under the peripheral control of other organs, we dissected and cultured fat bodies in Schneider’s *Drosophila* medium. Interestingly, the isolated larval fat bodies displayed no ICWs except for a few cells that sustained damage during dissection and maintained an abnormally high Ca^2+^ level (Fig. 1D). These data suggest that fat body ICWs are triggered by signals from other organs. To identify the specific organ responsible for this regulation, we co-cultured fat bodies with different larval organs including muscle/cuticle, intestine, and brain (Fig. 1C). ICWs were only triggered when fat bodies were co-cultured with brains (Fig. 1. C–E, **Supplementary video 2**), suggesting that these waves are initiated by a brain-derived factor.

To identify the putative factor, we generated a brain-conditioned medium by incubating Schneider’s *Drosophila* medium together with dissected larval brains (10 dissected 3rd instar larval brains in 150 μl medium), and applied the conditioned medium to isolated fat bodies. The brain-conditioned medium induced robust ICWs (Fig. 1F–G). To determine the nature of this factor, we filtered the brain-conditioned medium through a Pierce 10K MWCO concentrator that removed molecules larger than 10 kDa. The filtrate evoked robust fat body ICWs, suggesting that the factor has a relatively small molecular weight (Fig. 1G). We then treated the conditioned medium with proteinase K (0.1 mg/mL), DNAse I (1 U/mL) or RNAse A (0.1 μg/mL), followed by removal of added enzymes with the 10 kDa filter. Among these treatments, only proteinase K significantly reduced Ca^2+^ activities, suggesting that the factor is a small peptide (Fig. 1H).

### AKH released from APCs is responsible for inducing ICWs in the fat body

**Figure 2:**
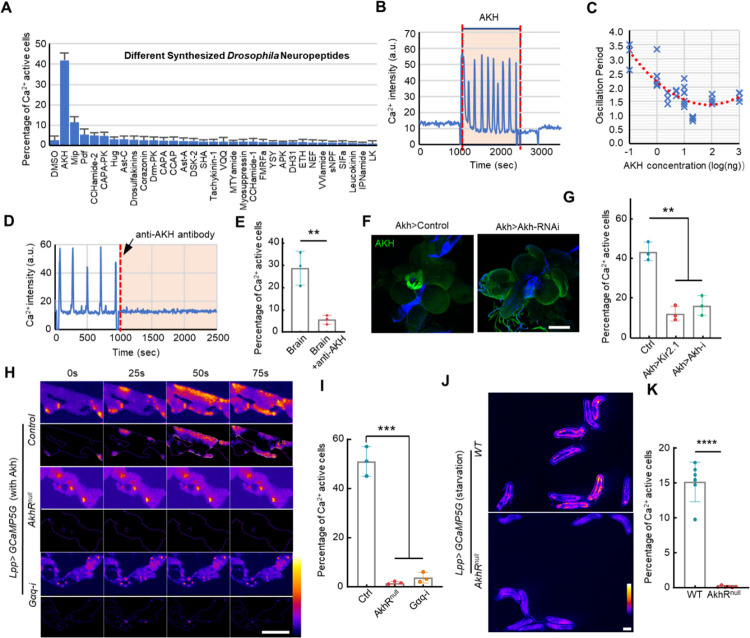
Brain-derived AKH is responsible for the ICWs in the fat body. (**A**) Quantification of Ca^2+^ activities triggered by different synthesized fly neuropeptides (0.1 ug/mL) in cultured larval fat bodies expressing GCaMP5G. (**B**) Ca^2+^ activities were monitored in the cultured fat bodies. AKH (0.1 ug/mL) was added to the dissected fat body, incubated for 15 min, and then washed away. (**C**) Relationship between the period of Ca^2+^ oscillation and concentration of applied AKH. (**D-E**) Ca^2+^ activity triggered by brain-conditioned medium was blocked by the addition of anti-AKH antibody (1:100 dilution). (**F**) AKH was efficiently knocked-down in APCs by *Akh*>*Akh-RNAi*. (**G**) Conditioned medium derived from brains with APCs silenced by Kir2.1 or with *Akh* knockdown showed a significantly reduced ability to trigger Ca^2+^ activity in the fat body. (**H-I**) Cultured fat body with *AkhR* mutant or *Gαq* knockdown failed to respond to AKH. The dynamic Ca^2+^ activities are highlighted in the lower panel by removing the constant background signal. (**J-K**) Ca^2+^ waves in free-behaving larvae are significantly reduced in *AkhR* mutant larvae. Data were plotted as mean ± SD. Scale bars, 200 μm (**F**), 500 μm (**H**), 1000 μm (**J**).

To identify the relevant peptide(s), we screened 32 major fly peptides on isolated fat bodies (10 ng/mL each). Among them, only AKH evoked significant Ca^2+^ waves in the fat body (Fig. 2A). Washing away the added AKH resulted in an immediate abrogation of ICWs, indicating that the Ca^2+^ waves require a sustained supply of AKH (Fig. 2B). The observation that the fat body responds within several seconds to the addition or removal of AKH, and the fact that there was no activity reduction in response to prolonged activation (up to 4 hours), suggests that the AKH receptor in the fat body has fast binding and dissociation kinetics with no signal adaptation. Further, we found that the fat body is sensitive to AKH at doses as low as 0.1 ng/mL, which is within the physical range of neuropeptides. In addition, both the amplitude and frequency of Ca^2+^ oscillations generally increased with AKH concentration (Fig. 2C, **Supplementary Fig. 3A-D**). Finally, knocking down the only y-encoded AKH receptor, *AkhR*, also reduced the Ca^2+^ oscillation frequency, suggesting that the frequency is regulated by the concentration of both ligand and receptor (**Supplementary Fig. 3E-F**).

To test whether AKH is indeed the fat-stimulating factor in the brain-conditioned medium, we added an anti-AKH antibody in the medium to sequester free AKH. Strikingly, the ICWs were blocked immediately after adding the anti-AKH antibody (Fig. 2D–E). Next, as AKH is secreted from the APCs of the ring gland, which is associated with the larval brain, we knocked down *Akh* in the APCs (*Akh-Gal4* > *UAS-Akh-RNAi*) or inhibited AKH release by expressing the neuronal silencing Kir2.1 potassium channel (*Akh-Gal4* > *UAS-Kir2.1*). This treatment suppressed the release of AKH resulting in a significant decrease in fat body Ca^2+^ waves in the co-culture experiment (Fig. 2F–G). Finally, fat bodies from *AkhR* mutant larvae no longer responded to the applied AKH peptide (Fig. 2H–I, **Supplementary Video 3**) and the Ca^2+^ waves were completely blocked in AkhR mutant larvae *in vivo* (Fig. 2J–K, **Supplementary Video 4**).

AkhR is a GPCR receptor that has been found to trigger both cAMP and Ca^2 + 21^. AKH is known to regulate Ca^2+^ increase by activating phospholipase C (PLC) and subsequent generation of IP3 and DAG^28^. To block the AKH-induced Ca^2+^ increase, we knocked down *Gαq*, which is responsible for the GPCR-dependent PLC activation. Knocking down *Gαq* in fat bodies blocked the AKH-triggered ICWs, suggesting that Gαq is an AKH-downstream effector for Ca^2+^ activity (Fig. 2H–I). Consistent with this observation, *Gαq* overexpression generated active Ca^2+^ waves both in isolated fat bodies without the addition of AKH and in free-behaving larvae on a 5% m/v sucrose diet (**Supplementary Fig. 4**). In addition, knocking down *SERCA*, an important ER-located Ca^2+^ pump, led to a sustained elevation of Ca^2+^ in the fat body (**Supplementary Fig. 4**). Collectively, these findings show that Ca^2+^ activity in the larval fat body is triggered by AKH secreted from the APCs, and relies on Gαq-mediated efflux of Ca^2+^ from the ER downstream of the AKH receptor.

### Global and local ICWs form through distinct mechanisms

**Figure 3:**
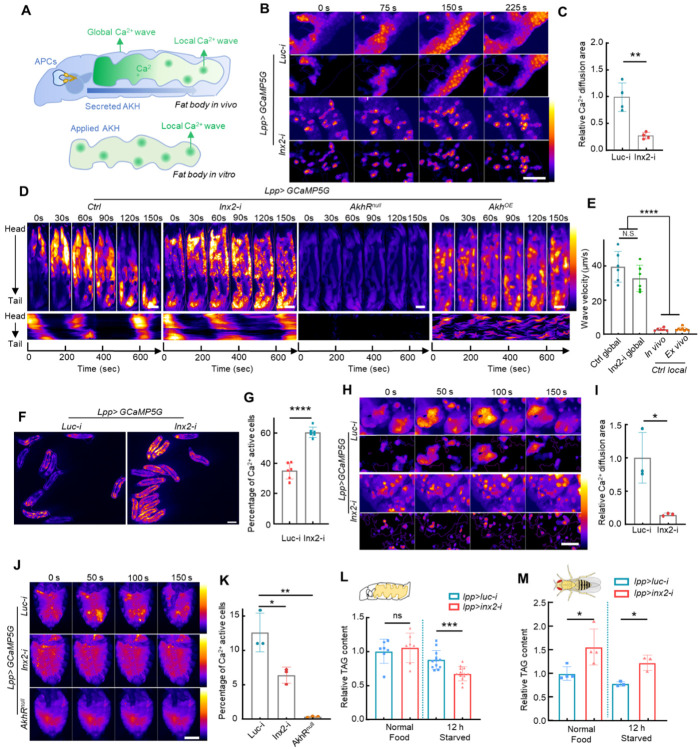
Global and local ICWs are regulated by gap junctions differently. **(A)** Both global and local ICWs are present in the larval fat body. Tissue-level ICWs traveling from larval head to tail are only observed *in vivo*. Conversely, local ICWs, initiated and propagated randomly in fat body cells, are evident in both *in vitro* and experimental settings. (**B-C**) Disruption of intercellular gap junctions by *Inx2* knockdown significantly scattered the Ca^2+^ activities in *ex vivo* cultured fat body. The dynamic Ca^2+^ activities were highlighted in the lower panel by removing the constant background signal. (**D-E**) *Inx2* knockdown does not affect the magnitude or period of global Ca^2+^ waves *in vivo*, while in *AkhR* mutants both global and local Ca^2+^ waves are completely blocked. Meanwhile, overexpressing AKH in the fat body led to a random propagation of Ca^2+^ waves. Larvae were kept under starved conditions to trigger the release of endogenous AKH. (**E**) Quantification of the traveling velocity of the global and local Ca^2+^ waves. (**F-G**) The proportion of cells with Ca^2+^ activities in the fat bodies of free-behaving WT and *Inx2 RNAi* larvae was quantified. (**H-I**) *Inx2* knockdown completely scattered the Ca^2+^ waves in the dissected adult fly fat bodies. The dynamic Ca^2+^ activities were highlighted in the lower panel by removing the constant background signal. (**J-K**) Adult female flies of the indicated genotype were starved for about 30 min and then glued alive on a glass slide with the ventral abdomen imaged. Ca^2+^ activities indicated by fat body-specific expression of *GCaMP5G* were monitored and quantified. (L-M) Effects of gap junction disruption in fat bodies during the larval and adult stages on TAG metabolism. Data were plotted as mean ± SD. Scale bars, 200 μm (**B, H**), 500 μm (**D, J**), 1 mm (**F**).

The AKH-induced ICWs observed in the fly fat body resemble the glucagon-triggered Ca^2+^ waves observed in the mammalian liver^1^. However, the biological significance of the ICW propagation in both mammalian liver and fly fat body has not been explored. Moreover, AKH triggers random local Ca^2+^ waves in the cultured fat body, but induces directional global ICWs propagating from the larval head to tail. The mechanism underlying the different behaviors of ICWs *in vivo* and *ex vivo* is unknown (Fig. 3A). To investigate the biological mechanism of ICWs propagation, we first genetically blocked the intercellular signal transduction in the fat body. Based on previous studies showing that the ICWs are mediated either by gap junctions or extracellular ATP^18,29^, we added ATP to the cultured fat body and found that it did not trigger ICWs (**Supplementary Fig. 5**), ruling out ATP as a trigger for ICWs in this system. We then screened all major gap junction proteins by RNAi and found that knockdown of Inx2 or Inx3 significantly reduced the intercellular Ca^2+^ waves in *ex vivo* fat bodies (Fig. 3B–C, **Supplementary Fig. 6**). Our observation is consistent with previous studies suggesting that Inx2 is predominately required for gap junction function in the fly epithelium^26,29,30^ and that Inx3 probably functions together with Inx2 to form a heterohexamer^31^. Both RNAi lines for *Inx2* and *Inx3* have been validated in multiple studies^30,32–34^ and showed similar phenotypes in our study. Since Inx2 knockdown exhibited the strongest phenotype, we used *Inx2-RNAi* to disrupt gap junctions in all subsequent experiments.

*In vitro* experiments demonstrated that *Inx2* knockdown effectively blocked the local propagation of Ca^2+^ activities (Fig. 3B–C, **Supplementary Video 5**). However, *in vivo* global ICWs were surprisingly unaffected by *Inx2* knockdown. Both propagation speed and oscillation period of global ICWs in the *Inx2-RNAi* fat body are similar to the control, while random local ICWs were blocked by the *Inx2-RNAi* (Fig. 3D–E, **Supplementary Fig. 7A**). In fact, Ca^2+^ oscillations in *Inx2-RNAi* flies exhibited an increase in amplitude and a decrease in frequency (**Supplementary Fig. 7B-D**). The overall effect of *Inx2* knockdown led to an unexpected elevation of average Ca^2+^ activity in the fat body of free-behaving larvae (Fig. 3F–G). To rule out the possibility of non-specific effects of *Inx2-RNAi*, we tested the effects of the gap junction inhibitor carbenoxolone. Consistent with *Inx2-RNAi*, carbenoxolone treatment showed a similar increase of Ca^2+^ amplitude and decrease of frequency in the cultured fat body (**Supplementary Fig. 7E-G**).

Despite these changes, our data clearly demonstrate that the spreading of the global ICWs *in vivo* does not require intercellular signal diffusion. Meanwhile, both the directional global and the random local Ca^2+^ waves were completely abrogated in *AkhR* mutants, suggesting that these Ca^2+^ waves in the fat body are triggered by extracellular AKH (Fig. 3D, **Supplementary Video 6**). Thus, we propose that local Ca^2+^ waves are generated by diffusion of Ca^2+^ from cells stochastically activated by AKH, but that the global Ca^2+^ waves are generated in response to the pulsed secretion and diffusion of a high level of extracellular AKH from the APCs near the larval head region (Fig. 3A). Supporting this hypothesis, the directional propagation of the ICWs were disrupted into a random pattern after overexpression of AKH in the fat body (Fig. 3D, **Supplementary Video 6**). In addition, we noticed that the *in vivo* global ICWs travel approximately 10 times faster than the local Ca^2+^ waves both *in vivo* and *ex vivo* (Fig. 3E). These data further support the notion that global ICWs are triggered by fast traveling extracellular AKH signals rather than by slow diffusion of Ca^2+^ spikes facilitated by gap junctions.

As most previous studies showed that ICWs in fly imaginal discs are significantly reduced after gap junction knockdown^24,26^, we wondered if the gap junction independent ICWs are stage-specific. Thus, we analyzed the Ca^2+^ activity in the adult fat body and discovered that the cultured adult fat body exhibited a similar gap junction-dependent local ICWs in response to the administered AKH like the larval fat body (Fig. 3H–I, **Supplementary Video 7**). However, contrary to the larval fat body, no global ICWs were observed in the adult fat body *in vivo*, and the local ICWs in the adult fat body were signi cantly reduced in adult flies with *Inx2* knockdown (Fig. 3J–K, **Supplementary Video 8**). These data indicate that the ICWs in the adult fat body behave as the previously reported Ca^2+^ waves in the imaginal disc epithelium, suggesting that global ICWs are unique to the larval stage.

As the increase of AKH secretion and cytosolic Ca^2+^ levels have been linked with TAG lipolysis in adipose tissues^21–23,35^, we assessed the effect of gap junction disruption on TAG catabolism under both normal feeding and starvation conditions. TAG levels in *Inx2* knockdown larvae remained unaffected under standard feeding conditions, as AKH secretion is not expected to be induced (Fig. 3L). However, under starvation conditions, TAG levels in gap junction knockdown larvae were significantly lower than in control larvae (Fig. 3L). This is consistent with our observation that gap junction blockage increases AKH-induced Ca^2+^ activities in larvae. In contrast, adult flies with disrupted gap junctions in the fat bodies displayed a significant increase in TAG accumulation under normal diet and starvation conditions (Fig. 3M), consistent with the reduction of Ca^2+^ activities in the adult fat body. Thus, the strikingly different responses of larval and adult fat bodies to gap junction disruption likely reflect the presence of global ICWs in larvae.

### Global ICWs are triggered by the periodic release of AKH in the circulating hemolymph

**Figure 4:**
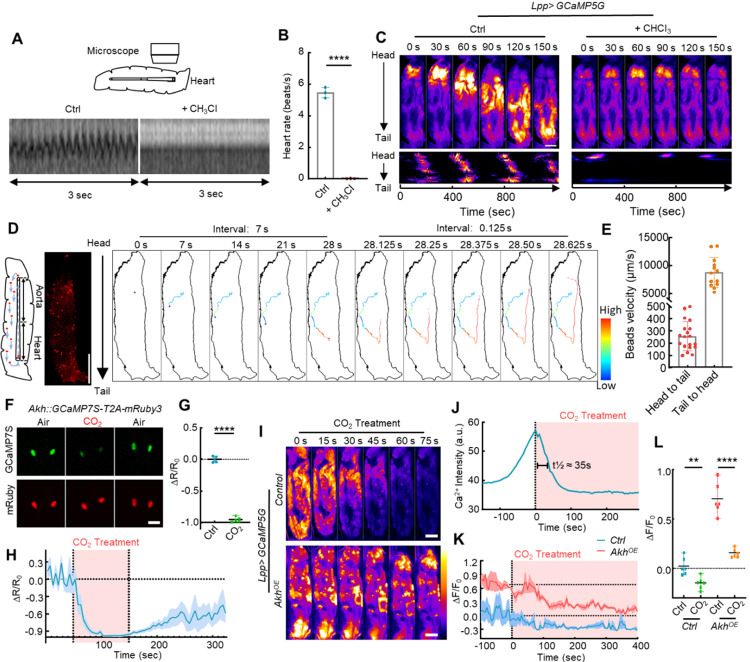
Global ICWs are generated by the periodic release of AKH in the circulating hemolymph. (**A-B**) 3^rd^ instar larvae were immobilized on a glass slide and imaged before and after the application of chloroform. Filter paper containing ~10 ul chloroform was placed close to the larvae, which temporarily stopped the heartbeat of the larvae for about 30 min. (**C**) Ca^2+^ imaging of the larvae treated with and without chloroform. (**D**) Fluorescent beads were injected into 3^rd^ instar larvae and traced under a microscope. The trajectory of one traced bead is shown with velocity coded in color. (**E**) The velocities of beads transported in the anterograde direction (from head to tail) and retrograde direction (from tail to head) were quantified. (**F-H**) 3^rd^ instar larvae were immobilized on a glass slide which is placed at the center of a CO_2_ fly anesthetizing pad. Neuron activity in the APCs was revealed using *Akh::GCaMP7S-T2A-mRuby3*. Ca^2+^ activities before and after CO_2_ application were quantified. (**I-L**) The Ca^2+^ waves of fat bodies are rapidly (t½ ≈ 35s) and completely blocked by CO_2_ treatment, while fat bodies overexpressing Akh still elicit Ca^2+^ waves when treated with CO_2_. (**M**) Simulated global ICWs in the fat body of WT and *Inx2-RNAi* larvae. Data in were plotted as mean ± S.E.M (**H, K**), or mean ± SD (others). Scale bars, 500 μm (**C, I**), 1000 μm (**D**), 50 μm (**F**).

To test whether global ICWs are triggered by the fast transport of extracellular AKH released from the APCs, we used chloroform to temporarily stop the heartbeats of 3rd instar larvae (Fig. 4A–B). Stopping the heartbeat completely prevented the propagation of global ICWs, and only the head region of the larvae showed periodic increase of Ca^2+^ activities (Fig. 4C). Notably, the global Ca^2+^ waves also showed a much longer period (~ 400 sec) than the intrinsic Ca^2+^ oscillation period in the fat body (~ 200 sec), supporting that the period of global waves is controlled by the pulsatile release of AKH from the APC cells rather than an intrinsic property of fat body.

We also noticed that the propagation speed of the global ICWs is around 40 μm/sec, which is more than 10 times faster than the intrinsic speed of the triggering wave of about 2.5μm/s (i.e. the speed of the local waves, which is generated presumably by gap-junction mediated intercellular signaling). This speed also surpasses the free diffusion velocity of a 1 kDa molecule (equivalent to the size of AKH) in water by approximately two orders of magnitude^36^ (Fig. 3E), which further supports that AKH released from APCs are transported by the circulating hemolymph. However, the flow characteristics of the fly hemolymph have never been measured before. Thus, we injected red uorescent polystyrene beads with a 5 μm diameter into the 3rd instar larvae and estimated the flow speed of the hemolymph by tracing the beads (Fig. 4D). After tracing multiple beads, we found that the anterograde (head to tail) flow speed of hemolymph is about 250 μm/sec and that the retrograde (tail to head) flow speed in the heart tube is nearly 10,000 μm/sec (Fig. 4E). This high-speed hemolymph circulation is fast enough to facilitate the transport of secreted AKH to the head region. Interestingly, the hemolymph flow in adult flies was primarily retrograde outside the adult heart tube, with an occasional reverse flow direction as previously reported^37^ (**Supplementary Fig. 8**). As the APCs are located in the anterior region of the adult thorax^21^, we speculate that the change in circulation direction of the adult hemolymph may make the transport of secreted AKH less efficient and thus be responsible for the absence of global ICWs.

Moreover, the circulating AKH model also implies that the release of AKH must have a short half-life compared with the 300 sec period of the global ICW, otherwise, AKH will accumulate in the hemolymph and trigger continuously random ICWs as observed in the *ex vivo* tissue culture. One way to test this model is to acutely block the release of AKH in APCs and monitor the decrease of Ca^2+^ in the fat body as a read-out of circulating AKH *in vivo*. Unfortunately, the available neuron-silencing optogenetic tools are incompatible with GCaMP imaging. As an alternative, we found that anesthetizing the larvae with carbon dioxide (CO_2_) rapidly suppressed the Ca^2+^ activity of the APC neurons, while the whole brain activity was largely unaffected (Fig. 4F–H, **Supplementary Fig. 9**). As our model predicted, the ICWs in the larval fat body rapidly returned to baseline within ~ 70 seconds after CO_2_ administration, implying that the *in vivo* functional half-life of AKH is ~ 35 seconds (Fig. 4I–L). Meanwhile, in larvae with AKH overexpression in the fat body, the significant level of ICWs remained after CO_2_ treatment, suggesting that CO_2_ does not block the fat body response to AKH (Fig. 4I–L).

### Computational modeling of the global and local ICWs

With the measured dynamic parameters of ICWs and AKH, we applied a receptor-operator calcium model to gain insights into the distinct dynamics of the ICWs in both larval and adult flies. The model incorporates three key elements: intracellular Ca^2+^ dynamics, intercellular signaling via gap junctions, and tissue-level AKH transport. The cytoplasmic Ca^2+^ level is governed by a receptor-operator calcium channel model^24,38^. When AkhR binds to AKH, it activates a downstream signaling pathway involving inositol trisphosphate (IP3), triggering a rapid Ca^2+^ efflux from the endoplasmic reticulum (ER) to cytoplasm via a positive feedback loop. The SERCA pump on the ER membrane acts as a slow negative feedback loop, restoring Ca^2+^ balance by pumping it back into the ER from the cytoplasm. Intercellular Ca^2+^ signaling through gap junctions is described by diffusion proportional to the cytoplasmic Ca^2+^ concentration difference between neighboring connected cells^24^. Either AKH binding to AkhR or the Ca^2+^ flow through gap junctions can trigger the intracellular fast-activation-slow-inhibition Ca^2+^ signaling cycles. In larvae, AKH transport and diffusion are described by a reaction-diffusion-advection equation. This equation accounts for the pulsatile AKH secretion from APCs and its uniform degradation. The advection reflects the transport of AKH via the circulating hemolymph. In adult flies, the global AKH pulses are absent, and the model only considers AKH diffusion with fluctuations. This distinction accounts for potential differences in extracellular AKH concentration dynamics between larvae and adult flies. Model details can be found in **Supplementary Methods 1**. The parameters used in the model are listed in **Supplementary Table 3**.

**Figure 5:**
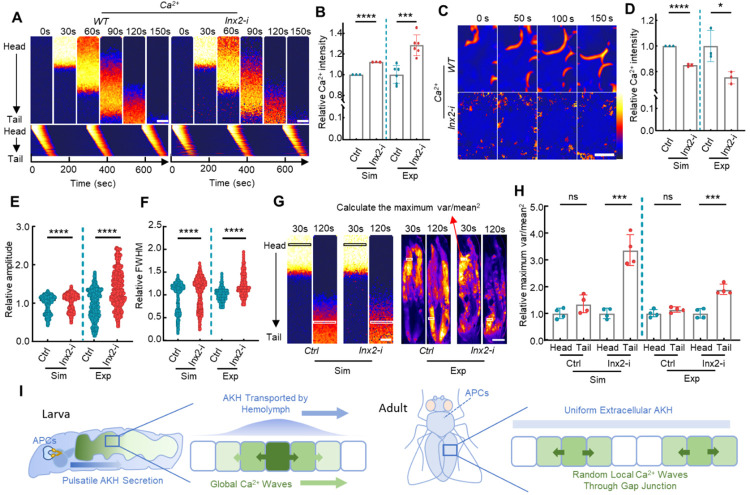
Computational modeling of the ICWs in larval and adult fiies. (**A**) Simulated global ICWs in the fat bodies of WT and *Inx2-RNAi* larvae. (**B**) Quantification of the relative Ca^2+^ intensity in the entire fat body using simulated (Sim) and experimental (Exp) data. The variance in the simulated data originates from the incorporation of random Ca^2+^ fluctuation into the model. (**C**) Simulated ICWs in the fat body of WT and *Inx2-RNAi* adult flies. (**D**) Quantification of the relative Ca^2+^ intensity in the entire fat body using simulated (Sim) and experimental (Exp) data. (**E-F**) Quantification of the relative amplitude and FWHM (full width at half maximum) of the Ca^2+^ signal in individual fat cells from WT or *Inx2-RNAi* flies using simulated (Sim) and experimental (Exp) data. (**G-H**) The correlation of Ca^2+^ activities along the wavefront was quantified by dividing the maximum variance of Ca^2+^ intensity by the square of the mean intensity. The relative maximum variations at the head and tail region were compared in both WT and *Inx2-RNAi* flies. (**I**) A schematic model of the ICWs in larvae and adult flies. The strong AKH pulse synchronized the global ICWs in the larva, which renders the signal diffusion through the gap junction less important. In contrast, in adult fly, the effective AKH is probably more uniform and triggers gap-junction-dependent random ICWs. Data were plotted as mean ± SD. Scale bars, 500 μm.

The simulations successfully replicated the observed differences in wave speed between larvae and adult flies, as well as the impact of gap junction knockdown on Ca^2+^ signaling (Fig. 5A–D). Both computational simulation and experimental data support that directional transport of AKH through the lymphatic circulation is responsible for the global ICWs with wave speed around 40 μm/sec, while the trigger wave mechanism through gap junctions is responsible for the local ICWs with wave speed around 3–4 μm/sec in WT and *Inx2-RNAi* flies^39^ (Fig. 5A–D, **Supplementary Video 11,12**). Meanwhile, the modeling also showed similar changes in average Ca^2+^ intensity and peak width in individual fat cells after gap junction knockdown in larval fat bodies (Fig. 5E–F), supporting that the modeling can precisely recapitulate the dynamics of Ca^2+^ activities.

Interestingly, our modeling results implied that the excitable Ca^2+^ signaling cycles are sensitive to both external and internal noises, such as fluctuations in AKH concentration or intracellular signaling molecules. These fluctuations have the potential to cause spontaneous Ca^2+^ signaling randomly, leading to asynchronous firing between individual cells. However, gap junctions help coordinate these excitation processes, resulting in spatially synchronized oscillations. In our simulations, we noticed that the Ca^2+^ wavefront is more synchronized in the WT larvae compared to larvae with the *Inx2* knockdown, despite similar wave speeds. To quantify the spatial coordination of Ca^2+^ activities, we calculated the normalized variance of Ca^2+^ intensity in the wavefront region (Fig. 5G–H). As the global ICWs propagate from head to tail, the normalized variance remains low for WT larvae but increases sharply in larvae with gap junction knockdown. Notably, the experimental data also exhibited a similar increase of variance in the tail region of the *Inx2-RNAi* larvae but not in the WT larvae (Fig. 5G–H). This finding reinforces the relevance of our modeling results, although the biological significance of the variance increase requires further exploration.

### Regulation of AKH release from APCs by amino acids

**Figure 6:**
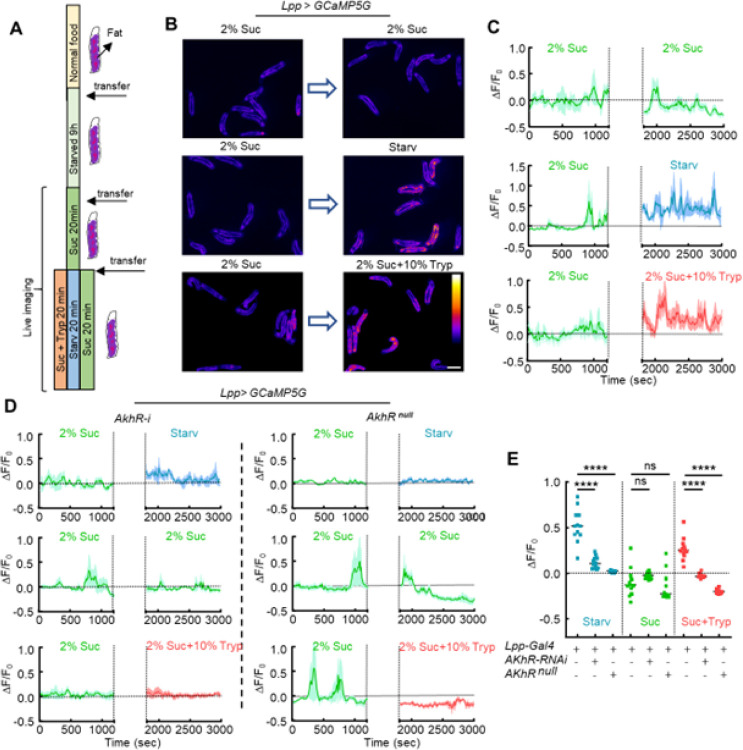
AKH-dependent ICWs are regulated by dietary sugar and protein. (**A**) Early 3^rd^ instar fly larvae were transferred onto 2% agarose for 9 hours and then onto 2% agarose + 2% sucrose for 20 min. These conditioned larvae were further transferred onto different test foods containing 2% sucrose, 2% sucrose + 10% Tryptone (protein diet), or no nutrient (starvation diet). (**B-C**) Representative Ca^2+^ images and Ca^2+^ activities of free-behaving larvae transferred between different diets. Ca^2+^ activities were calculated from the average signals obtained from all imaged larvae. The gap between the two diets is the larvae transfer time which is ~10 min. (**D**) Representative Ca^2+^ activities in the fat body of free-behaving larvae with whole-body *AkhR* mutant or fat-specific knockdown of *AkhR*. (**E**) Quantification of Ca^2+^ activities in the fat body of free-behaving fly larvae. Data in were plotted as mean ± S.E.M (**C, D**), or mean ± SD (**E**). Scale bars, 2 mm (**B**).

Our findings demonstrate that the fat body exhibits a specific and rapid response to extracellular AKH. This property provides a clear and direct readout for monitoring real-time secretion of AKH in free-behaving larvae under different nutrient conditions. As most previous live-imaging setups used immobilized or even anesthetized larvae, which may cause undesirable distress and artefacts, we decided to study metabolic signaling in free-behaving animals. In addition, previous studies have suggested that AKH, a functional homolog of mammalian glucagon, is released upon starvation^3,5,35,40^. Early 3rd instar larvae were first starved for 9 hours to cleanse their digestive systems, and then fed on 2% sucrose for 20 min, allowing the fat body Ca^2+^ activities and AKH secretion to return to a basal level (Fig. 6A). Subsequently, these conditioned larvae were transferred onto 2% agarose plate with 2% sucrose, 2% sucrose plus 10% Tryptone (a protein-rich diet equivalent to approximately 5% protein), or 2% agarose with no nutrient (starvation diet). Larvae fed on a starvation diet displayed a significant increase in fat body Ca^2+^ waves after ~ 10 minutes of feeding, indicating a swift response to starvation, which agrees with the established function of AKH as a starvation-induced hormone. Importantly, consumption of the protein-rich diet also significantly increased Ca^2+^ activities in the fat body within just 10–15 minutes (Fig. 6B–C, **Supplementary Video 13**), suggesting that AKH secretion is also triggered by amino acids. To confirm whether the protein-induced fat body Ca^2+^ waves are indeed AKH-dependent, we compared WT larvae with larvae carrying a *AkhR* mutation or fat-body specific AkhR knockdown using *Lpp-Gal4*. In both cases, we observed a significant reduction of the Ca^2+^ waves under both starvation and protein-feeding conditions, supporting the idea that both processes depend on AKH signaling (Fig. 6D–E).

**Figure 7:**
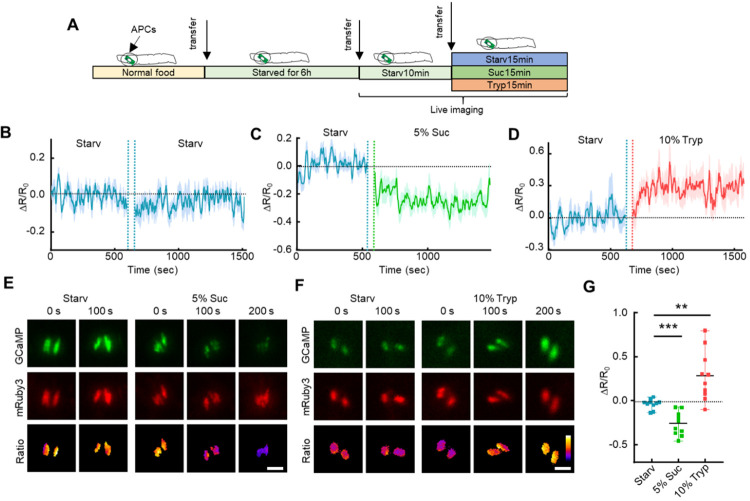
AKH secretion from the APCs is regulated by amino acids. (**A**) 1^st^ instar fly larvae were transferred onto 2% agarose for 6 hours and then onto different test foods containing 5% sucrose, 10% Tryptone (protein diet), or no nutrient (starvation diet). (**B-D**) Representative Ca^2+^ activities in the APCs of free-behaving larvae transferred between different diets. The gap between the two diets is the larvae transfer time which is ~10 min. (**E-F**) Representative Ca^2+^ images of the APCs of free-behaving larvae fed on different diets. (**G**) Quantification of Ca^2+^ activities in the APCs of free-behaving larvae. Data in were plotted as mean ± S.E.M (**B-D**), or mean ± SD (**G**). Scale bars, 25 μm (**E, F**).

The *in vivo* Ca^2+^ activity of the fat body suggests that AKH release from APCs is triggered by amino acids. To test this directly, we examined whether APC activity is regulated by a protein diet in free-behaving larvae. We used *Akh-Gal4* > *GCaMP5G-T2A-mRuby3* to visualize and quantify Ca^2+^ activity in the APCs. However, as the thick cuticle of the 3rd instar larvae caused a strong blurring of the signal from the APCs when they moved, we decided to use 1st instar larvae, which are smaller and more transparent. We used an Extended-Depth-of-Field (EDoF) microscope, which turns slow 3D imaging into a quick 2D acquisition (**Supplementary Fig. 10A-D**). In addition, because the Ca^2+^ signals from the APCs are much dimmer than those of the fat body, we used a starvation diet as the initial condition to achieve a reliable visualization of Ca^2+^ activity. 1st instar larvae were starved for 6 hours on 2% agarose plate, then transferred to new agarose plates containing 5% sucrose (sugar diet), 10% Tryptone (protein-rich diet), or 2% agarose only (starvation) (Fig. 7A). As expected, Ca^2+^ levels in the APCs significantly decreased after being fed on 5% sucrose (Fig. 7B–G, **Supplementary Video 6**). Interestingly, Ca^2+^ levels in the insulin-producing cells (IPCs) were reduced when we performed simultaneous dual labeling of both the APCs and IPCs (**Supplementary Fig. 10E-G**). This real-time observation of the counter activities of the IPCs and APCs strongly supports the reliability of this live-imaging system. Next, we tested the effect of a 10% tryptone diet and found that protein consumption triggers a Ca^2+^ increase in the APCs after ~ 10 minutes of feeding, which is consistent with the quick Ca^2+^ response observed in the fat body (Fig. 7B–G). We used 5% sucrose instead of 2% because 5% sucrose triggers a quicker and stronger AKH suppression. However, for the experiment in 3rd instar larvae, the secretion of AKH secretion is too severely suppressed with 5% sucrose, making the Tryptone diet less effective.

**Figure 8:**
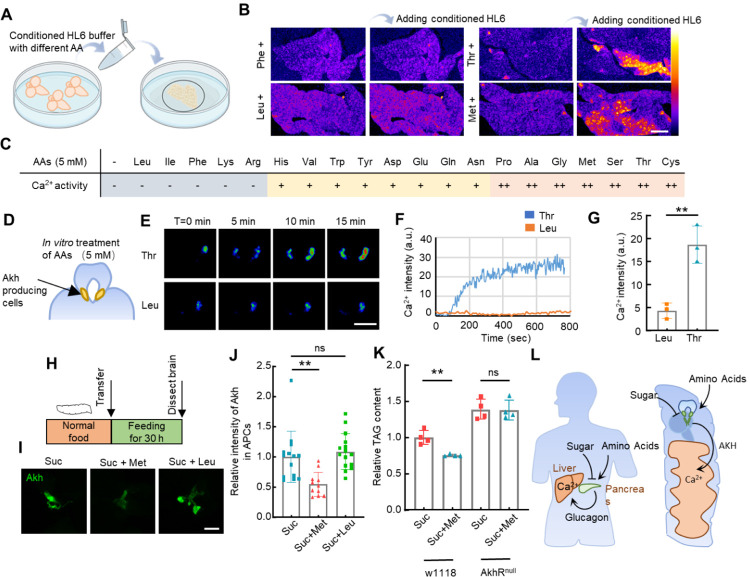
AKH secretion from APCs is regulated by amino acids. (**A**) Brain-conditioned HL6 media with each amino acid (5 mM) were generated and transferred to dissected 3^rd^ instar larval fat bodies. (**B**) Representative Ca^2+^ images of fat bodies before and after the addition of brain-conditioned HL6 media with the indicated amino acids. (**C**) Ca^2+^ activities in the bodies treated by brain-conditioned HL6 medium with the indicated amino acids. Percentage of fat body cells with positive Ca^2+^ signal: “++” means more than 10%, “+” means 10%−5%, “-” means less than 5%. (**D**) AKH-producing CC cells are located at the “foot” region of the ring gland associated with the larval brain. Ca^2+^ activity in the CC cells was monitored using *Akh-Gal4*>*GCaMP5G*. (**E-F**) Dissected larval brains together with ring glands were incubated in HL6 medium with amino acids. Representative Ca^2+^ activities in APCs after the addition of the indicated amino acids (5 mM) are shown. (**G**) Quantification of Ca^2+^ activities in the APCs 15 mins after the addition of the indicated amino acids. (**H**) 3^rd^ instar larvae fed on indicated food for 36 hours and then dissected. AKH levels in the APCs were stained with anti-AKH antibody. (**I-J**) Representative staining of AKH and quantifications of the fluorescent intensity in APCs of fly larvae fed on 2% sucrose, 2% sucrose + 40 mM Met, or 2% sucrose + 40 mM Leu. (**K**) TAG contents of adult flies fed on indicated foods for 48 hours were measured. Diets containing 2% sucrose or 2% sucrose + 40 mM Met were used. (**L**) Sugar and amino acid sensing properties of AKH as well as its downstream Ca^2+^ waves are functionally conserved compared with mammalian glucagon. Data were plotted as mean ± SD. Scale bars, 50 μm (**B**), 25 μm (**E, I**).

Studies in mammals have found that only some amino acids trigger glucagon release, with branch-chained amino acids failing to induce such secretion^41^. Thus, we tested which particular amino acids may activate AKH-mediated Ca^2+^ waves in *ex-vivo* fat body tissues using our conditioned-medium system. As the complete Schneider’s *Drosophila* medium contains all amino acids and triggers the release of AKH from dissected larval brains, we prepared a basal *Drosophila* HL6 buffer devoid of any amino acids (referred to as HL6(AA-) buffer). Subsequently, each amino acid (5 mM) was individually added to the HL6(AA-) buffer to assess its capacity to induce AKH release (Fig. 8A). Brain-conditioned HL6(AA-) buffer does not activate the cultured fat body; however, the addition of most small polar amino acids triggered AKH secretion and subsequent elevation of fat body Ca^2+^ (Fig. 8B–C). Notably, the large branch-chained amino acids leucine (Leu) and isoleucine (Ile) failed to trigger AKH release, akin to their effects on the mammalian glucagon system. We further monitored Ca^2+^ activities in APCs in isolated larval brains. APCs responded to threonine (Thr) within two minutes and reached an activation plateau at around six minutes, consistent with the response speed observed *in vivo*. In contrast, APCs showed little response to Leu (Fig. 8E–G). Finally, the release of AKH from APCs was confirmed by AKH staining of APCs in 3rd instar larvae. Starvation, known to trigger AKH release, served as a positive control (**Supplementary Fig. 11**). Next, we tested 36 hours of feeding on 5% sucrose, 5% sucrose plus 40 mM methionine (Met), or 5% sucrose plus 40 mM Leu (Fig. 8H–J). Met feeding significantly reduced the AKH signal in the APCs compared to the sugar control and Leu, suggesting that Met triggers the release of AKH *in vivo*. Previous studies have shown that increased AKH levels lead to lipolysis in fat body tissues, especially in adult flies^35^. Hence, we examined whether amino acid feeding could reduce triacylglycerol (TAG) content. Indeed, 40 mM Met feeding reduced the TAG content in adult flies, and this reduction was entirely abrogated in AkhR mutant animals (Fig. 8K). Altogether, our results show that specific dietary amino acids, i.e., Methionine and Threonine, are sensed by APCs to trigger AKH release, which in turn activates ICWs in the fat body.

## Discussion

In this study, we demonstrate that AKH secreted from the APCs stimulates ICWs in the *Drosophila* fat body to promote lipid metabolism. Furthermore, we discovered that the global and local ICWs in the fat body are generated through different molecular mechanisms: global Ca^2+^ waves are generated by extracellular circulation of AKH secreted from APCs in a pulsatile manner, whereas local Ca^2+^ waves are formed through intercellular signal propagation mediated by gap junctions. Finally, we found that specific dietary amino acids activate the APCs, leading to increased intracellular Ca^2+^ and subsequent AKH secretion.

### ICWs in the fly fat body are controlled by AKH-AkhR signaling

Previous studies have established Ca^2+^ as a key regulator of lipolysis in the fly fat body^22,23^, and AKH has been found to trigger Ca^2+^ increase in the fat body under *ex vivo* conditions^28^. Our current study demonstrates that the primary *in vivo* driver of Ca^2+^ activity in the fly fat body is the AKH-AkhR pathway and its downstream effector *Gαq*. Similar to mammalian glucagon, AKH is a central hormone that has been found to integrate diverse biological processes, including mobilization of lipid storage, stimulation of locomotion, oxidative stress protection, and immune response^21^. Therefore, it is important to study the release of AKH at high temporal resolution. Our findings suggest that Ca^2+^ signaling within the fly larval fat body serves as a reliable real-time indicator for AKH signaling in free-behaving animals.

### AKH-mediated ICWs in the larvae and adult fat body are driven by distinct mechanisms

Ca^2+^ has been found to generate a variety of inter and intra-cellular activities, including flashes, sparkles, oscillations and ICWs, which have been implicated in diverse biological processes^29^. In this work, we focus on ICWs in the *Drosophila* fat tissue. We demonstrate that these fat ICWs are specifically under the control of the AKH hormone produced from the brain-associated neuroendocrine cells, the APCs. Canonically, ICWs are considered to spread through tissues via gap-junctions^17,26,29,42^. However, we delineate a new gap-junction independent mechanism in which AKH release from the brain actively diffuses through the circulating lymph of the animal, which in turn results in the organ-level ICWs. These gap junction-independent Ca^2+^ waves uncovered in our study present an intriguing model illustrating how a hormone can function as an extracellular orchestrator, creating a collectively moving pattern across a large epithelial tissue. Our findings reveal that these gap-junction-independent ICWs in the larval fat body bypass the need for intercellular Ca^2+^ propagation and are self-sufficient in maintaining tissue-level Ca^2+^ activities. These tissue-level ICWs also suggest that AKH is secreted in a strong pulsatile manner in larvae, a phenomenon similar to the pulsatile release of mammalian glucagon and insulin, which are disrupted in patients with type-2 diabetes, potentially contributing to hyperglucagonemia^43,44^. However, the biological significance of this pulsatile hormone release compared to continuous release is not clear. Our study suggests that in the fly larva, a pulsatile secretion of AKH creates a strong increase of hormone “shock” that collectively activates fat body cells, which renders the intercellular Ca^2+^ spreading between neighboring cells less important.

Intriguingly, the adult fat body depends on gap junctions to uphold a functional Ca^2+^ level under starvation or amino acid feeding conditions. Meanwhile, no organ-level global Ca^2+^ waves were observed—the Ca^2+^ waves detected *in vivo* in the adult fat body appear completely random, suggesting that the circulating AKH in adult hemolymph may not be strong enough to collectively activate the fat body cells. Thus, in the absence of a strong extracellular AKH pulse, cells activated by AKH become sparsely distributed as in the adult fat body, requiring intercellular spreading of Ca^2+^. The biological significance behind these differences between larval and adult tissue remains an open question. Understanding these differences might help us understand the difference in response to AKH-AkhR signaling in larva vs. adult fly, i.e., AKH-AkhR signaling activation of lipolysis in the adult fly fat body but not in the larval fat body.

Through mathematical modeling of these waves in the fat body, we also noticed that inclusion of random fluctuation is essential to recapitulate the Ca^2+^ wave properties in fat bodies with *Inx2-RNAi*. The increase of Ca^2+^ activities after gap junction knockdown can be observed only when the random fluctuation of the Ca^2+^ concentration is considered in our model. Within the parameter range that ensures oscillating/wave behavior, an increase in Ca^2+^ fluctuation intensity will amplify the magnitude difference between WT and *Inx2-RNAi* in the simulation of the larval fat body. The precise reason why random fluctuations serve as a determining factor for wave properties requires further investigation, yet our results suggest that the random effect should not be overlooked in the research of biochemical waves.

### Dietary amino acid-mediated activation of APCs

The *in vitro* kinetics of Ca^2+^ activities also reveal intriguing aspects of amino acid sensing and AKH-AkhR signaling. We found that cultured APCs do not respond immediately to the applied amino acids but instead exhibit a gradual increase in activity over a 5–10 minutes period. This suggests that amino acids may not function through a rapid response mechanism such as ligand-gated channels, but via a comparatively slower metabolic process, possibly involving an increase in cytosolic ATP following amino acid breakdown. However, it remains to be elucidated whether the APCs sense amino acids directly or indirectly through other neurons or a secondary metabolite within the brain. Furthermore, the fast and continuous response of the fat body to extracellular AKH without adaptation implies that AkhR probably does not undergo activation-induced inactivation. This immediate and persistent response to extracellular AKH could provide flies with an advantage in promptly adapting their metabolic state to environmental changes. Meanwhile, the *in vivo* half-life of AKH is remarkably short due to an unknown mechanism, probably involving a specific secreted proteinase. Identification of this unknown serum factor may provide key insights into the dynamic regulation of hormonal signaling.

By tracing Ca^2+^ activities in the fly fat body and AKH-producing cells in response to different nutrients, we found that AKH secretion is regulated by certain dietary amino acids in the fly hemolymph, which subsequently increases the mobilization of fat body lipids through AkhR-Gαq signaling. Interestingly, previous studies have identified other amino acids sensing mechanisms in different organs: amino acid triggers the release of GBP1/2 and Stunted from the fat body to stimulate the insulin-like peptides secretion and promote larval growth^45,46^, essential amino acids promote the release of CNMa from intestine cells to regulate feeding behavior^47^, FMRFa secretion from brain neurons is triggered by amino acid consumption to mobilize lipid stores^48^, and insulin-producing cells (IPCs) sense amino acids to increase insulin-like peptides production^20^. It will be interesting to explore how and where these amino acid-dependent signals interact or integrate to achieve metabolic homeostasis.

Although we have observed that amino acids regulate the secretion of AKH, the precise biological significance of this phenomenon is still not fully understood. Our study primarily focused on the regulation of AKH secretion and its effect on fat body Ca^2+^ increase and subsequent TAG lipolysis. However, it is conceivable that the activation of AkhR has a more extensive role than facilitating neutral lipid mobilization. Recently, AKH has been found to activate extracellular signal-regulated kinase (ERK), which in turn increases amino acid catabolism and gluconeogenesis in the fly fat body^49^. Together with our observations, it seems plausible that amino acid-induced AKH secretion serves as a mechanism for flies to process excessive amino acids ingested from the diet. AKH is believed to be a functional homolog of mammalian glucagon, which is specifically produced under starved or low-energy conditions to promote lipolysis in peripheral organs. However, we found that AKH is not only stimulated under energy challenges conditions but also by a high protein diet. Similarly, a high-protein diet has also been reported to trigger mammalian glucagon. A more comprehensive study of the downstream signaling pathway of AKH is needed to fully understand the consequence of this AKH-mediated amino acid sensing axis. In addition, our and previous work suggest that different dietary amino acids can trigger different neuronal centers in the fly brain, i.e., leucine and isoleucine specifically trigger insulin-producing cells (IPCs) in the larval brain^20^, whereas we found that these two amino acids do not trigger APCs to release AKH. Further research is required to decipher how activation of different neuronal centers may take place following a high protein diet consisting of different groups of amino acids.

Lastly, the increase of amino acid uptake and usage by AKH signaling in the fat body resembles the recently discovered mammalian Liver-α-Cell axis, whereby an increase in glucagon levels upregulates the expression of specific amino acid transporters such as Slc38a4 and Slc38a5 in the liver, thereby enhancing amino acid uptake and promoting gluconeogenesis as well as urea production^50^. It will be interesting to investigate whether certain amino acid transporters are similarly upregulated by AKH in the fly fat body. Moreover, elevated amino acid levels in the bloodstream not only stimulate glucagon secretion but also contribute to α-cell proliferation, leading to pancreatic α-cell hyperplasia in mice, creating a lasting endocrine feedback^50^. Whether the function of APCs is modulated by amino acid consumption in *Drosophila* is still unknown. Experiments examining whether high protein intake induces lasting effects on APCs could provide evidence for the conservation of the Liver-α-cell axis across species.

## Data Availability

The data presented in this study are available on request from the corresponding author.
